# Intensive out-of-hospital coaching for frequently hospitalized COPD patients: a before-after feasibility study

**DOI:** 10.3389/fmed.2023.1195481

**Published:** 2023-10-17

**Authors:** Bart A. C. Noort, Taco van der Vaart, Jan van der Maten, Esther Metting, Kees Ahaus

**Affiliations:** ^1^Department of Operations, Faculty of Economics and Business, University of Groningen, Groningen, Netherlands; ^2^Department of Pulmonology, Medical Center Leeuwarden, Leeuwarden, Netherlands; ^3^Data Science Center in Health, University Medical Center Groningen, University of Groningen, Groningen, Netherlands; ^4^Department of Primary and Elderly Care, University Medical Center Groningen, University of Groningen, Groningen, Netherlands; ^5^Health Services Management and Organisation, School of Health Policy & Management, Erasmus University Rotterdam, Rotterdam, Netherlands

**Keywords:** COPD, out-of-hospital coaching, self-management, re-hospitalization, feasibility, real-life data

## Abstract

**Background:**

This study assesses whether out-of-hospital coaching of re-hospitalized, severe COPD patients by specialized respiratory nurses is feasible in terms of cost-effectiveness, implementation, and recipient acceptability. The coaching was aimed at improving patients’ health management abilities, motivation for healthy behavior, strengthening the professional and informal care network, stimulating physiotherapy treatment and exercise training, improving knowledge on symptom recognition and medication use, and providing safety and support.

**Methods:**

Cost-effectiveness of 6 months of out-of-hospital coaching was assessed based on a before-after intervention design, with real-life data and one-year follow-up. A total of 170 patients were included. Primary (questionnaires, meeting reports) and secondary data (insurance reimbursement data) were collected in one province in the Netherlands. The implementing and recipient acceptability was assessed based on the number of successfully delivered coaching sessions, questionnaire response rate, Patient Reported Experience Measure, and interviews with coaches.

**Results:**

Post-intervention, the COPD-related hospitalization rate was reduced by 24%, and patients improved in terms of health status, anxiety, and nutritional status. Patients with a high mental burden and a poor score for health impairment and wellbeing at the start of the intervention showed the greatest reduction in hospitalizations. The coaching service was successfully implemented and considered acceptable by recipients, based on patient and coach satisfaction and clinical use of patient-reported measures.

**Conclusion:**

The study demonstrates the value of coaching patients out-of-hospital, with a strong link to primary care, but with support of hospital expertise, thereby adding to previous studies on disease- or self-management support in either primary or secondary care settings. Patients benefit from personal attention, practical advice, exercise training, and motivational meetings, thereby improving health status and reducing the likelihood of re-hospitalization and its associated costs.

## 1. Introduction

Worldwide, more than 300 million people suffer from Chronic Obstructive Pulmonary Disease (COPD), of which up to 3 million die annually (1–3). In the Netherlands, almost 585,000 people are reported to suffer from this tobacco or air pollution-induced lung disease (4). Patients with COPD experience respiratory distress and often require hospitalization due to exacerbations, thereby reducing their quality of life and invoking significant healthcare costs (5, 6). Preventing exacerbations and subsequent hospitalizations remains challenging as, besides poor lung function, factors like anxiety, depression, poor disease-coping, and low self-esteem also play a role (7–9). Many patients have a relatively low socio-economic status and poor disease literacy, thereby complicating the goal of improving patients’ self-management skills and motivating them to adapt their behavior (10). As both formal and informal caregivers from multiple disciplines are usually involved in COPD treatment, improving care coordination is desirable (11, 12). The above outlines why researchers call for more research on exacerbation prevention, with particular attention to coaching, and in a real-life setting (13, 14). We respond to this call by presenting an out-of-hospital coaching intervention aimed at re-hospitalized COPD patients [Severity score D, based on the Global Initiative for Chronic Obstructive Lung Disease (GOLD) classification] (15), evaluated with real-life data.

Given the various psycho-social and physical complaints related to COPD, it is difficult to achieve actual improvements in health skills and behavior (9, 16, 17). COPD coaching interventions are aimed at providing additional support and teaching patients how to manage their disease (18). Through frequent sessions, a coach can build a trusting relationship, thereby providing safety, reducing stress, increasing confidence, and motivating patients’ active disease management (16, 17, 19). Due to the variability in COPD symptoms, patients have individual care needs, requiring inputs from various caregivers such as GPs, pulmonologists, nurses, psychologists, social workers, physiotherapists, and informal caregivers. By acting as a case manager, a coach can improve communication between caregivers, as well as between the patient and caregivers, and ensure the coordination of tasks and responsibilities so that patients receive the right care in the right place.

COPD coaching, and patient-centered care interventions in general, vary in terms of the coaching experience and expertise, and the location and frequency of coaching, showing the need for evaluating demarcated interventions (5, 20). In this study, we evaluate high-frequency, home-based coaching conducted by a specialized respiratory nurse. We posit that such personal and intensive support is important for patients who have been hospitalized due to a COPD exacerbation. Moreover, the knowledge and experience of the nurses can help patients gain a better understanding of their disease and improve their disease literacy, psychosocial wellbeing, and self-management. Although there are indications that coaching may indeed reduce exacerbations and hospitalizations (13, 14, 21–25), this has, to the best of our knowledge, not been studied in patients who have been hospitalized repetitively due to exacerbations and who receive intensive out-of-hospital coaching by experienced respiratory nurses (17, 19).

This paper presents an intervention on out-of-hospital coaching of COPD patients conducted in a province in the northern part of the Netherlands from 2016 to 2019. A total of 170 patients who were admitted for a second time because of a COPD exacerbation for a second time within a year were enrolled in the study and received 6 months of coaching from a specialized hospital respiratory nurse. The patients were followed by collecting real-life data regarding COPD symptoms, functional state, motivation and disease-management awareness, mental health, nutrition, and chain-wide care use and costs based on insurance reimbursement data. In this study, we first investigate *how hospitalizations, disease-related symptoms, wellbeing, motivation, and disease-management awareness develop over time* and, secondly, *whether the coaching intervention was implemented as planned and considered acceptable by coaches and COPD patients*. Answering these questions will resolve whether COPD patients that are predominantly treated in a secondary care setting can benefit from out-of-hospital, home-based support, and how this should be organized. Furthermore, this real-life study aims to extend the limited knowledge of how out-of-hospital coaching influences care use throughout the care chain.

## 2. Materials and methods

### 2.1. Study design and data collection

The study consists of three parts. In the first part, we conducted an intervention study in a real-life setting to evaluate changes in the health status of patients who participated in the out-of-hospital coaching intervention program which involved 7 home-based and 2 phone-based meetings. Using validated questionnaires, we gained insights into changes in COPD-related symptoms, wellbeing, and disease-management awareness. In the second part, a cost-effectiveness evaluation was carried out based on reimbursement data from the main health insurer in the studied province. We analyzed care use and costs throughout the care chain for a subset of participating patients from the first part of the study (henceforth referred to as the “insurance data” subset). Finally, we evaluated whether the out-of-hospital coaching program was successfully implemented as planned and considered acceptable by coaches and COPD patients. Herewith the study addresses three of the typical goals of feasibility studies, as classified by Bowen et al. (26): cost-effectiveness (efficacy), implementation, and recipient acceptability.

The study was conducted in a province of the Netherlands with around 500,000 inhabitants. The included patients received a one-year coaching intervention. We assessed the hospitalization rate of the patients from before to one year post-intervention.

### 2.2. Patient population and recruitment

From June 2016 to May 2018, hospitalized patients were recruited from the nursing wards of the participating hospitals. To be eligible to participate in this study, patients had to be at least 18 years old and hospitalized with a COPD exacerbation, be diagnosed with COPD, and been previously hospitalized for a COPD exacerbation within the previous year. Potential subjects with severe mental health problems who were therefore considered not coachable (determined by the pulmonologist) or for whom the pulmonologist regarded referral to an intramural revalidation clinic necessary (following the current hospitalization) were excluded. Patients were screened by the coaches, if necessary in consultation with a pulmonologist, on their suitability to participate. Eligible patients were informed about the study by one of the coaches, received verbal and written information, were asked to sign a consent form, and had a more elaborate introductory meeting while still hospitalized. Each patient was followed up for two years. Here, we present data for the first follow-up year. The study protocol was approved by the regional medical ethical committee (#NL54328.099.15). The study was registered in the Netherlands Trial Register (NTR5624).

### 2.3. Design of the coaching intervention

The intervention was part of a collaboration between medical specialists operating in four hospitals, GPs who represent a major provincial primary care group, coaches (respiratory nurses) and physiotherapists. Before the intervention started coaches attended two training sessions provided by external professional trainers to obtain the required additional knowledge and ensure consistency in the coaching procedure. The first training session focused on motivational interviewing and psychosocial support. The second training was aimed at improving disease literacy, health-related behavior, and practical issues related to the personal and home situation of COPD patients.

Patient coaching was performed by 14 experienced respiratory nurses (13 female and 1 male with secondary vocational or higher professional education) based in one of the four hospital’s pulmonology nursing wards. Once a patient was discharged, the following sessions were scheduled: home-coaching session 1 (after 1–4 days), session 2 (after 2 weeks), sessions 3–7 (every 4 weeks), session 8 (phone call, after 7 months), session 9 (phone call, after 1 year) (see Supplementary material file 1).

The main goals for the coaches were established in a coaching protocol: (1) educate the patient to improve their health literacy, (2) strengthen knowledge and motivation for healthy behavior, (3) strengthen the patient’s personal network, and (4) facilitate coordination of care among different providers. During the coaching sessions, the coaches discussed the patient-reported outcomes with the patient. Discussion of each coaching goal had to be checked in an online registration system (CastorEDC). Coaches actively encouraged patients to participate in physical exercise and breathing training by a COPD-specialized physiotherapist, which was financially supported for non-insured patients. Coaches further coordinated care delivery by mapping the patient’s care needs and communicating with and/or referring to other care providers such as the GP, district nurse, or dietician.

### 2.4. Measurements

#### 2.4.1. Intervention study: patient-reported symptoms, wellbeing, and disease-management awareness (all patients)

After each meeting, a summary was written in the patient’s personal file and in the online registration system. The coaches furthermore noted data regarding weight and smoking status (pack-years). In advance of each meeting, certain of the following questionnaires were completed by the patient (online or on paper) and the outcomes were discussed with the patient: Medical Research Council (MRC) (27), Clinical COPD Questionnaire (CCQ) (28, 29) St George Respiratory Questionnaire (SGRQ) (30), Hospital Anxiety and Depression Scale (HADS) (31–33), Patient Activation Measure (PAM) (34, 35) and the Short Nutritional Assessment Questionnaire (SNAQ) (36) (see also Supplementary material file 1). If the patient was unable or not sufficiently literate to complete the questionnaires, this task was completed together with the coach during the meeting.

#### 2.4.2. Evaluation of reimbursement data: care use and costs based on insurance data (subset)

Reimbursement data was available for 85 of the 170 included patients (50%) as they were insured by the health insurer involved in this study. These data consist of occurrences and costs of COPD-hospitalizations, and other care use (outpatient visits, GP visits, ambulance services, physiotherapy). The numbers and costs of systemic corticosteroids, inhaled corticosteroids, inhaled bronchodilators, and fixed-dose combination were determined from the reimbursement data. Oral corticosteroid maintenance treatment was defined as treatment of more than 5 days involving doses of 2.5, 5, 10, or 20 mg. High-dose oral corticosteroid treatment was defined as treatment of 5 days or fewer, with a dose of 20 mg or higher. To calculate cost-effectiveness, we established the costs of the intervention, based on the number and duration of coaching meetings, administration time, transportation costs, training, and IT.

### 2.5. Sample size calculation and statistical analysis

#### 2.5.1. Sample size

Based on the reviewed literature, we conservatively expected an intervention effect of a 12.5% reduction in hospitalization rate (37–41) Considering previous findings, we considered a participation rate of 80% as realistic (38, 39, 42) Comparable studies report drop-out rates of 1.9–13.7%, and so we estimated a conservative drop-out rate of 20%. Using the estimated intervention effect, of a 12.5% reduction in hospitalization rate, an alpha level of 5%, and a beta level of 20%, a required sample size of 80 was calculated with a paired two-tailed *T*-test (SPSS). As the hospitalization rate was established using reimbursement data for a subset of the patients (an estimated 50% of the total population), the minimum number of participants was set at 160.

#### 2.5.2. Statistical analysis of health status, care use, and costs

Two panel linear regression models were developed (R-Studio):

(1) A random-effects model with each questionnaire score as a dependent variable. As independent variables, these models included time after intervention (6 and 12 months post-intervention), age, sex, drop-out (survival and quit), and insurer type. The baseline questionnaire score was included as a control variable. The group in the insurance data subset was compared with the total data set to see if the populations were comparable. The scores from meeting 10 (two years post-intervention) were not included as too few data had been collected at the time of analysis.
(2) A random-effects model with the costs of hospitalizations and hospitalization rate as the dependent variable. These models included time post-intervention, before vs. post-intervention, physiotherapy treatment (yes/no), hospitalization rate or costs before the intervention, age, BMI (normal weight, underweight, overweight), patient-reported symptoms, wellbeing, and disease-management awareness at the start of the intervention, sex, and drop-out (survival and quit) as independent variables (see Supplementary material file 2 for details on the models).

### 2.6. Evaluation of implementation and acceptability

The implementing and recipient acceptability of the out-of-hospital COPD coaching intervention was assessed by analyzing the following aspects: (1) the Proportion of delivered coaching sessions; (2) The patient response rate to questionnaires; (3) Patient care evaluation based on Patient Reported Experience Measure (PREM–Chronic care version, see Supplementary material file 1) (43); and (4) Care-giver evaluation based on a qualitative evaluation study.

The caregiver evaluation consisted of structured interviews with six of the participating coaches, conducted by one of the coaches and an independent researcher (report available upon request, written in Dutch).

## 3. Results

### 3.1. Health status evaluation (all patients)

#### 3.1.1. Inclusion and baseline characteristics

**FIGURE 1:**
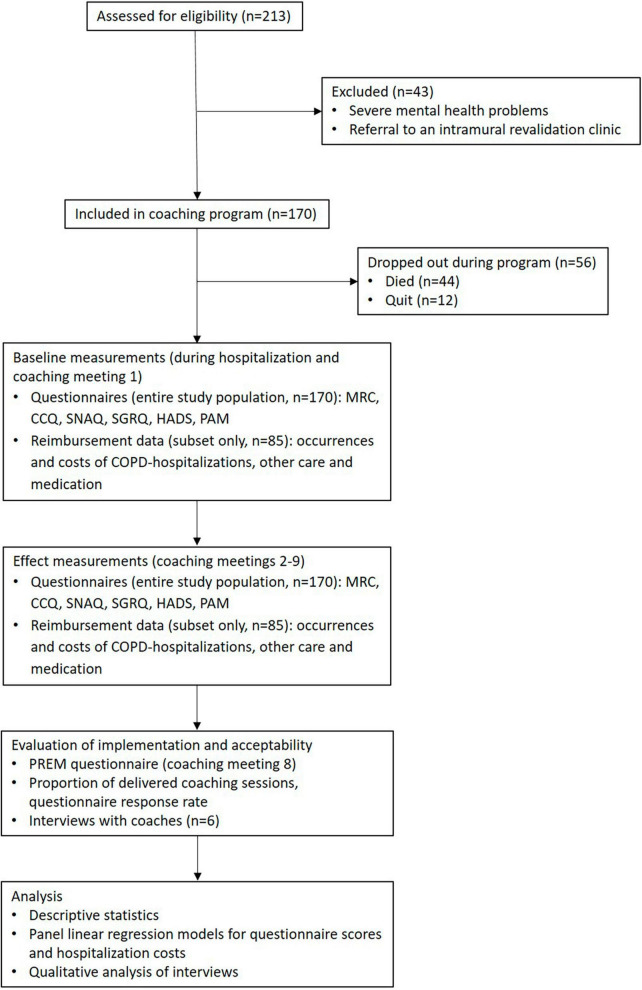
Flow diagram of patient inclusion, data collection and analysis.

Between June 2016 and May 2018, 170 patients [average age 69 (SD: 9.6), 90 (52.9%) female] were enrolled in the study. This total amounted to 80% of patients who met the inclusion criteria. The main reasons for not participating were concern about the time or mental burden of the coaching sessions. Table 1 shows the baseline characteristics of the participating patients, for the total patient group and for the insurance data subset. During the study, 44 (25.9%) patients died, and 12 (7.1%) patients quit. The main reasons for quitting during the study were a significant deterioration in physical status, or finding it an excessive time or mental burden. Figure 1 provides a flow diagram of patient inclusion, data collection, and analysis.

**TABLE 1:** Baseline characteristics at start of the intervention, for all patients and for insurance data subset.

Number of patients	All patients	Insurance data subset
**Participants**	***n* (%)**	***n* (%)**
Hospital 1	69 (40.6)	39 (45.9)
Hospital 2	33 (19.4)	18 (21.2)
Hospital 3	28 (16.5)	12 (14.1)
Hospital 4	40 (23.5)	16 (18.8)
Total	170	85
**Comorbidity**	***n* (%)**	***n* (%)**
Diabetes	20 (11.8)	10 (11.8)
Cardiovascular disease	51 (30.0)	25 (29.4)
Malignity	19 (11.2)	9 (10.6)
Sleep apnea	7 (4.1)	5 (5.9)
Osteoporosis	14 (8.2)	5 (5.9)
Obesity	6 (3.5)	4 (4.7)
Kidney failure	2 (1.2)	1 (1.2)
No comorbidity	36 (21.2)	19 (22.4)
Unknown	15 (8.8)	7 (8.2)
**Other characteristics**	***n* (%)**	***n* (%)**
Sex	90 (52.9) females	42 (49.4) females
Currently smoking	31 (18.2)	14 (16.5)
Died during study	44 (25.9)	20 (23.5*)*
Quit during study	12 (7.1)	7 (8.2)
Physiotherapy	unknown	72 (85.9)
	**Mean (SD)**	**Mean (SD)**
Age (years)	69 (9.6)	69 (9.8)
BMI (kg/m^2^)	25.7 (5.8)	25.8 (6.6)
**Patient-reported outcomes**	**Mean (SD)**	**Mean (SD)**
MRC	4.0 (1.1)	4.1 (1.2)
CCQ	3.1 (1.0)	2.7 (0.9)
SGRQ	61.5 (13.7)	63.9 (13.0)
HADS anxiety	6.8 (4.2)	6.6 (4.1)
HADS depression	7.5 (4.3)	7.4 (4.3)
PAM	53.3 (11.6)	53.1 (12.1)
SNAQ	1.5 (1.5)	1.5 (1.5)

#### 3.1.2. Patient-reported symptoms, wellbeing, and disease-management awareness

Table 2 shows a statistically significant improvement 6 months post-intervention compared to the baseline in terms of CCQ (before: 3.1, post: 2.8, *p* < 0.05) and SNAQ scores (before: 1.5, post: 0.9, *p* < 0.01) (based on the panel linear regression model, see also Supplementary material file 2A). However, these improvements did not reach the minimum clinically important difference (MCID) threshold. Six months post-intervention, 20.6% (MRC questionnaire) and 45.4% (CCQ questionnaire) of the patients reported a clinical improvement in at least one of the patient-reported symptoms, wellbeing, and disease-management awareness scores. For the CCQ (before:3.1, post:2.8, *p* < 0.05), HADS (anxiety) (before: 6.8, post: 6.0, *p* < 0.05), and SNAQ (before: 1.5, post: 0.8, *p* < 0.01) questionnaires a statistically significant improvement over baseline was also found 12 months post-intervention. However, these improvements again failed to reach the MCID threshold. Twelve months post-intervention, 19.6% (MRC) and 40.2% (CCQ and SGRQ) of the patients showed a clinical improvement in at least one of the patient-reported symptoms, wellbeing, and disease-management awareness categories. The baseline CCQ (*p* < 0.01), SGRQ (*p* < 0.01), and HADS depression (*p* < 0.05) scores of patients who died during the follow-up period were significantly worse than those of survivors. Comparing the scores of the insurance data subset with the total patient dataset showed a significant difference (*p* < 0.05) in terms of the CCQ questionnaire, with the subset scores being worse than the total data set scores.

**TABLE 2:** Outcomes of questionnaires regarding symptoms, wellbeing, and disease-management awareness for all patients.

Questionnaire	Start (*n* = 170)		6 months post intervention (*n* = 141)			12 months post intervention (*n* = 92)		
	**Score Mean (SD)**	**Surv vs. died Mean (SD)**	**Score Mean (SD)**	**Clinically improved *n* (%)**	**Surv vs. died Mean (SD)**	**Score Mean (SD)**	**Clinically improved *n* (%)**	**Surv vs. died Mean (SD)**
MRC	4.0 (1.1)	3.8 (1.2)	4.0 (1.2)	29 (20.6)	3.8 (1.2)	3.9 (1.2)	18 (19.6)	3.7 (1.2)
		4.4 (1.0)			4.4 (1.0)			4.4 (0.8)
CCQ	3.1 (1.0)	3.2 (1.0)	2.8 (1.1)*	64 (45.4)	2.7 (1.1)	2.8 (1.0)*	37 (40.2)	2.6 (1.0)
		3.0 (0.9)**			3.2 (1.0)			3.5 (1.1)
SGRQ	61.5 (13.7)	59.1 (13.9)	59.9 (13.2)	53 (37.6)	58.1 (13.8)	59.6 (14.0)	37 (40.2)	57.6 (14.2)
		68.5 (10.1)**			66.6 (8.1)			68.4 (9.8)
HADS Anxiety	6.8 (4.2)	7 (4.2)	6.5 (4.6)	48 (34.0)	6.5 (4.8)	6.0 (4.7)*	37 (40.2)	5.8 (4.4)
		6.5 (4.1)			6.4 (4.1)			7.0 (6.0)
HADS Depression	7.5 (4.3)	7.7 (4.5)	7.2 (4.1)	47 (33.3)	6.9 (4.1)	7.2 (4.3)	42 (45.7)	6.8 (4.2)
		7.3 (3.6)*			8.0 (4.0)			9.0 (4.7)
PAM	53.3 (11.6)	53.1 (11.2)	55.0 (11.6)	51 (36.3)	55.9 (11.6)	52.7 (11.4)	29 (31.5)	52.5 (10.1)
		54.3 (13.2)			53.6 (11.8)			54.3 (16.7)
SNAQ	1.5 (1.5)	1.3 (1.4) 2.0 (1.7)	0.9 (1.2)**	52 (36.9)	0.8 (1.2) 1.1 (1.3)	0.8 (1.2)**	30 (32.6)	0.7 (1.3) 1.1 (0.9)

Number (n) clinically improved indicates the number of patients whose score improved by at least the MCID. Scores are indicated for patients who died during the follow-up period (*n* = 44), vs. survivors (surv). Six months post-intervention, 15 patients had died and 9 had quit, and data were not available for 5 patients. Twelve months post-intervention, 44 patients had died, 12 had quit, and data were not available for 22 patients. Asterisks indicate a statistically significant difference compared to at the start based on panel linear regression (*p < 0.05; **p < 0.01) (see Supplementary material file 2A).

### 3.2. Cost-effectiveness evaluation (insurance data subset)

#### 3.2.1. COPD-related hospitalizations

**FIGURE 2:**
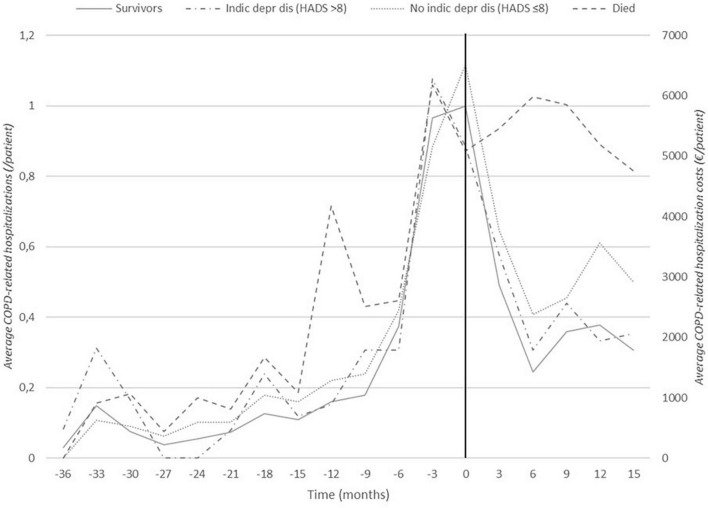
Frequency of hospitalizations due to COPD exacerbations: 36 months before to 15 months after the intervention (insurance data subset). Black vertical line indicates start of the intervention at t0. Distinctions are made between patients who eventually died during the follow-up period (this group consists of 21 patients, of which 13 had died 15 months post-intervention) and survivors and between patients with (HADS-depression >8) and without (HADS-depression ≤8) depression at the start of the intervention.

Figure 2 shows the hospitalizations rate due to COPD exacerbation from 3 years prior untill 15 months after the start of the coaching intervention for patients in the insurance data subset. The annual COPD-related hospitalization rate for patients in the insurance data subset reduced by 24% from 2.39 one year before the intervention (*n* = 85, SD: 1.15) to 1.81 one year post-intervention (*n* = 69, SD: 2.16) (*p* < 0.01 based on the panel linear regression model for both hospitalization rate and costs, see also Supplementary material file 2B). As shown in Table 3, the average hospitalization rate was 0.57 (*n* = 84, SD: 0.87) in the period from one year to 6 months before the intervention and increased to 1.82 (*n* = 85, SD: 0.89) in the 6 months immediately before the intervention. After the start of the individual coaching trajectory, the patients were hospitalized on average 1.09 times (*n* = 80, SD: 1.36) in the first 6 months post-intervention and 0.83 times (*n* = 69, SD: 1.21) in the following 6 months.

**TABLE 3:** The number of delivered services or care products and costs per care category (standard deviation).

	−1 to −0.5 year (*n* = 84)	−0.5 to 0 year (*n* = 85)	0 to + 0.5 year (*n* = 80)	+ 0.5 to +1 year (*n* = 69)
	**Mean (SD)**	**Mean (SD)**	**Mean (SD)**	**Mean (SD)**
Hospitalizations	0.57 (0.87) €4,116 (€8,794)	1.82 (0.89) €9,341 (€6,416)	1.09 (1.36) €5,732 (€7,853)	0.83 (1.21) €5,491 (€12,413)
Ambulance services	1.49 (3.57) €420 (€757)	3.3 (6.51) €969 (€1,114)	2.24 (5.18) €577 (€732)	2.72 (6.24) €649 (€1,013)
Outpatient visits	0.75 (0.69) €249 (€334)	0.31 (0.51) €74 (€128)	0.93 (0.71) €354 (€433)	0.69 (0.68) €194 (€260)
GP visits	23.02 (8.15) €287 (€217)	25.91 (11.46) €376 (€301)	24.76 (9.04) €359 (€323)	22.53 (10.77) €341 (€445)
Physiotherapy visits	7.98 (16.04) €234 (€526)	8.44 (16.52) €254 (€512)	14.20 (16.92) €459 (€635)	14.33 (17.51) €477 (€613)
**Systemic corticosteroids**				
Maintenance dose	90.4 (135.50) €4.54 (€5.79)	69.03 (110.64) €2.47 (€4.22)	117.87 (165.01) €5.65 (€16.67)	118.85 (167.05) €5.88 (€17.30)
High-dose/exacerbation	1.24 (1.46) €1.45 (€1.98)	2.15 (1.85) €2.41 (€2.17)	1.92 (1.92) €2.00 (€2.13)	1.47 (1.57) €1.73 (€1.99)
**Inhaler medication**				
Inhaled corticosteroids	7.43 (48.13) €23.16 (€77.42)	9.78 (56.26) €30.95 (€95.15)	9.79 (51.13) €37.70 (€156.36)	13.74 (64.06) €51.98 (€180.89)
Inhaled bronchodilators^∧^	139.92 (235.49) €220 (€167)	132.12 (221.78) €185 (€149)	130.90 (203.48) €173 (€138)	119.16 (187.57) €150 (€133)
Combination inhalers^#^	63.94 (158.79) €267 (€193)	111.48 (255.16) €280 (€195.66)	145.09 (258.08) €271 (€184)	178.10 (278.93) €285 (€203)
Total	€5,900	€11,917	€8,136	€7,841

Data are only shown for patients with a minimum of 100 days of data per half-year period. For the periods 0 to + 0.5 year and + 0.5 to + 1 year, data were extrapolated if the patient had dropped out of the study. ^∧^Inhaled bronchodilators: long- and short-acting beta2 agonists, long- and short-acting muscarinic agonists. ^#^Combination inhalers: inhaled corticosteroids and bronchodilators.

Figure 2 illustrates these findings and shows that, for patients with a high mental burden at the start of the intervention, the hospitalization rate strongly reduced after coaching (HADS depression >8: before: 2.58, SD: 0.90, post: 1.58, SD: 1.53). This pattern significantly differs from that of patients without a high mental burden (HADS depression ≤8: before: 2.65, SD: 1.32, post: 1.86, SD: 2.43, *p* < 0.05, for both hospitalization rate and costs). The hospitalization rate also more strongly decreased for patients with an initial high SGRQ score (SGRQ ≥ 65: before: 3.00, SD: 1.33, post: 1.78, SD: 2.09) than with those with a low SGRQ score (SGRQ < 65, before: 2.29, SD: 0.96, post: 1.76, SD: 2.24, *p* < 0.05, for hospitalization costs only). The average annual COPD-related hospitalization rate was fairly stable in the group of patients who eventually died during the follow-up period (before: 2.86, SD: 1.38, post: 2.31, SD: 3.12, ns) while there was a significant decrease in the surviving patients (before: 2.51, SD: 1.15, post: 1.37, SD: 1.38, *p* < 0.05, for hospitalization rate only).

#### 3.2.2. Care use and costs

The reimbursement data from the insurance company provides chain-wide care use and costs (Table 3). The reduced hospitalization rate equates to an annual cost reduction of €2241 per patient in the first year post-intervention (sum of hospitalization costs t-1 year to t0, minus the sum of hospitalization costs t0 to t + 1 year). In line with this, we see a decrease in the costs for ambulance services of €163. Costs of physiotherapy services and outpatient visits increase by €448 and €225, respectively. Other types of GP, hospital, home nursing, and mental care (not shown in the data) remain fairly stable over time. Concerning pharmaceutical use, we observed increases in maintenance doses of systemic corticosteroids, inhaled corticosteroids, and combination inhalers, albeit with high variability. Balancing the reduced care costs with the increased outpatient and physiotherapy costs suggests a cost reduction of on average €1,731 per patient per year. The coaching intervention costs are calculated at €900 per patient per year: coaches spent on average 18.5 h per patient (coaching meetings: 9 h, travel time: 7 h, administration time 2.5 h = €630 at an hourly rate of €35). Other costs per patient per year were, IT costs: €35, training: €25, project management and support: €150, and supervision by pulmonologist: €60.

Deducting the intervention costs from the cost reduction realizes a cost-saving of €830 per patient per year, meaning that the coaching intervention is cost-positive over a one-year follow-up period.

### 3.3. Implementation and acceptability

A total of 170 patients [90 females (52.9%)] average age 69 years (SD: 9.6) received 1274 home coaching sessions, of which only one session was missed (canceled by the patient). The response rate to the questionnaires, both used clinically and for research purposes, was 87.1%. The main reason for not completing the questionnaires was the administrative burden experienced by patients. Based on the PREM questionnaire, patients rated the coaching positively, with an average score on all 15 items of 4.3 (1–5 scale, SD: 1.25). Patients were especially positive about openness (4.7, SD: 0.4) clear explanation (4.7, SD: 0.6), advice (4.4, SD: 1.0), shared decision-making (4.0, SD: 1.5), expertise (4.7, SD: 0.49), and effectiveness (4.2, SD: 1.1). The “alignment between professionals” item was rated lower (3.6, SD: 1.7). On the general evaluation question, “I would recommend this service to other patients,” patients gave an average score of 8.7 (1–10 scale, SD: 1.1).

The qualitative evaluation conducted by one of the coaches further showed that all six interviewed coaches highly appreciated the coaching intervention. Analysis of the interviews indicated care improvements in four domains (see Table 4 for illustrative quotes): (1) Caregiver-patient relationship—a better trust-based relationship was felt important to gain a better understanding of the various problems of the patient; (2) Collaboration—the coaching improved collaboration between different care providers, although this also appeared challenging due to time pressures on care professionals and limited facilities for information exchange; (3) Self-management support—the greater attention given to patients’ psycho-social problems, palliative care, and disease coping behavior was seen as essential for this patient group; and (4) Professional development—the coaches acquired greater insight into the perceptions of patients, and also of the primary care providers. Further, the coaches could see the benefit of further developing motivational interviewing, mental care, and post-hospitalization care.

**TABLE 4:** Illustrative quotes on each of the established domains on which the coaches experienced improved care delivery (based on qualitative evaluation).

Domain	Illustrative quote
Care giver-patient relationship	Particularly because the patient knows you are from the hospital, that feels very familiar to them. You know exactly how they feel, we know what it takes, a lot of people around the patient don’t know that—coach 5
	I think because he knew me, and I had met him four times…I met him in the hospital and I just had to tell him “you are not ok, right?,” and he poured out his heart, purely because as a coach you have his trust, then you can mean so much—coach 1
Collaboration	You experience that there is a need for someone who coordinates, who knows the situation both at home and in the hospital—coach 2
	Of course you signal things but, at a certain point, you have to take your hands off. It is still their own responsibility, the goal is self-management—coach 3
Self-management support	They often still have questions about their disease. And that is not just respiratory-related, it relates to their multiple problems, like heart failure and diabetes. So, it is more about being ill as a whole—coach 2
	When I visited her for the second time, she said: “I don’t want to be hospitalized again,” I am ok with dying, I want to make a good plan for how to proceed—coach 2
Professional development	The added value is that you get an understanding of the patient’s personal situation, then you can signal why things go wrong at home and why they are hospitalized so often—coach 3
	I would like more training on self-management support, there is a lot I still don’t know—coach 4

Overall, the implementation of the coaching intervention appeared feasible. Practical matters that needed to be organized were the implementation of an online registration system for the questionnaires (use of tablets and the CastorEDC system), and financial compensation for coaches’ time and travel costs. Coaches experienced the registration system as useful for administrative and coaching purposes. Although patients could complete the questionnaires online, paper versions were still widely used as patients appeared to have limited digital literacy. Training of the coaches and the coaching itself was experienced as successful in terms of professional development and added value for the patient. A remaining organizational problem was the limited staff availability, despite the financial compensation. Also, coaches experienced a high administrative burden.

## 4. Discussion

### 4.1. Main findings

Our findings indicate that patients may benefit from intensive out-of-hospital coaching in terms of wellbeing and COPD-related hospitalizations. The intervention proved feasible in terms of implementing the coaching service, increased referral to and use of physiotherapy, the clinical use of patient-reported symptoms, wellbeing, and disease-management awareness questionnaires. It was also cost-effective and received high patient and coach satisfaction ratings. The average annual hospitalization rate was reduced by 24%, from 2.39 (*n* = 85, SD: 1.15) to 1.81 (*n* = 69, SD: 2.16) for the insurance subset for which we had reimbursement data. We also found statistically significant, albeit not clinically relevant, improvements in respiratory symptoms (CCQ), mental wellbeing (HADS-anxiety), and nutritional status (SNAQ).

### 4.2. Comparison with previous findings

Suissa et al. (44) showed that the likelihood of a subsequent exacerbation leading to hospitalization increases threefold after a second exacerbation and 24-fold after the tenth, relative to the first. This progressive character of COPD and the increasing risk of hospitalization indicates that the successful implementation of an out-of-hospital coaching service with a subsequent reduction in COPD-related hospitalizations may indeed be beneficial for patients with severe COPD. The fact that the steep decrease in hospitalization rate continues up to one year post-intervention supports this conclusion. Our result is in line with other studies that show a reduction in hospital admission risk of between 15 and 63% (13, 14, 23, 24, 45, 46). However, several other studies show no reduction, or even a small increase in hospitalizations (47–51). The most likely explanation for these different findings is that coaching is not always conducted by specialized, experienced respiratory nurses, but sometimes by more generally trained nurses or medical assistants (19). The coaches in our study were able to provide high-quality care and advice on prevention and the effects of exacerbations and gave significant attention to end-of-life care. Moreover, while most reported studies concern stand-alone interventions, the current study was executed in a real-life setting, thereby providing continuity of care, and strengthening caregiver-patient relationships.

### 4.3. Susceptibility to coaching

The improvement in the anxiety score (+ 0.8 compared to baseline) and the strong reduction in the hospitalization rate for patients with a poor initial depression score indicates that the effectiveness of coaching is dependent on a COPD patient’s mental burden. This supports the assumption that personal, face-to-face, and continuous attention by an experienced, familiar nurse is a prerequisite for success (52, 53). Specifically targeting psychological counseling at patients with mental health problems may prevent hospitalizations, while patients with predominant somatic problems may benefit more from pharmacologic treatment or pulmonary rehabilitation (7, 8, 54). Importantly, irrespective of mental status, we found that patients with a high baseline score for physical impairment and wellbeing (SGRQ) benefit from coaching, which is promising given the severity of the disease in the participating patients. Thus, GOLD-D COPD patients are likely to benefit from coaching in their home environment. Nevertheless, most research into COPD hospitalization prevention is still focused on treatment in a hospital (17). In line with other studies, our findings call for increased attention to and capacity for treating COPD patients outside the hospital system (55–57). In our study, the reimbursement data indicated that there was no increase in GP care despite the increased use of physiotherapy care. This confirms the conclusion from our qualitative evaluation, as well as from other scholars, that collaboration and alignment between caregivers should receive greater attention in future initiatives aimed at improving chronic disease management (12, 58). At the same time, we recognize that this will require providing sufficient time for care professionals and a supportive system for information exchange.

### 4.4. Patient-reported symptoms, wellbeing, and disease-management awareness

In contrast with several other studies, the patients participating in this trial had on average an improved COPD-related functional state (CCQ) both 6 months and one year post-intervention (59, 60). The average CCQ score of patients who died during the follow-up period was significantly higher than that of survivors. As such, a worsening CCQ score may be an important indicator of likely exacerbations and even death, showing the clinical value of this measure (29, 61). Overall, we would urge further use and evaluation in a clinical setting of patient-reported measures so that exacerbations can be signaled promptly and emergency hospitalizations prevented. Patients also showed improvements in terms of anxiety complaints, as reported elsewhere by Bucknall et al. (48) but not by other studies (50, 60, 62) suggesting a need for further research. Concerning nutritional status, patients appeared to suffer less from undernutrition, the main cause of muscle waste (63, 64). This seems to be a “quick-win” when aiming to improve disease management skills, probably because it is straightforward to explain. Moreover, the negative effect of obesity on COPD patient health further stresses the importance of attention to nutrition and exercise by a coach (65, 66). Concerning motivation for, and awareness of, the patient’s influence on improving their health-related behavior, we found no sustained improvement. Other studies confirm the challenge of measuring and achieving a sustained improvement in motivation, treatment adherence, and activation of COPD patients (67–70).

### 4.5. Strengths and limitations

In this study, we have chosen a before-after intervention design based on real-life data, an approach that has its advantages but also limitations. It is likely that one effect seen in our study, an increase in hospitalizations followed by a decrease, could be due to a regression to the mean (71, 72). To what extent this is the case could be assessed by using a control group for comparison purposes. Unfortunately, this was not possible in this study due to the limited number of available participants. Nevertheless, the hospitalization history of our patients shows a very low initial hospitalization rate, followed by a progressive pattern. It is well-known that the risk of exacerbations occurring after the first exacerbation increases almost threefold, and then keeps on increasing (44). Following the start of the coaching intervention, this pattern was broken and the lower hospitalization rates were still evident one year post-intervention when our analysis ended. The retrospective use of reimbursement data proved valuable to follow COPD disease progression, in line with the study of Jiang et al. (73).

Around 25% of the patients died during the follow-up period which, given the average age and disease status, is in line with earlier research that shows a mortality rate of around 23% one year after initial hospitalization (44, 74). However, this means that a significant part of the sample population could not be followed up, which may influence our findings. Indeed, patients who died during the follow-up period scored worse overall on CCQ, SGRQ, HADS-depression, and had a higher average COPD-related hospitalization rate. Hence, we should not attempt to draw any causal conclusions on the effect of the out-of-hospital coaching intervention. Nevertheless, we can provide in-depth, real-life insights into the organization and experience of coaching for severely ill COPD patients. Treating secondary care patients in an out-of-hospital setting while using patient-reported measures has only been limitedly reported, and hence our study provides new knowledge for both research and practice.

The patients in the insurance data subset scored worse than the total patient group for CCQ. We failed to establish any systematic medical, insurance-related, or socio-demographic reason for this difference, thereby limiting the comparability of the two groups. This difference might lead to an underestimation of the effect of the coaching intervention.

### 4.6. Future implications for research and practice

This study highlights the important role of specialized respiratory nurses, operating in the home setting of discharged COPD patients. Practitioners should consider the value of providing support by a healthcare professional who is acquainted with the patient but also has sufficiently specialized knowledge of COPD and is able to coach the patient. Given the substantial capacity requirements, eHealth support should be considered in future coaching interventions, but without diminishing the role of experienced and highly educated nurses. The quantitative and qualitative findings further show the challenge of providing a more integrated way of COPD care delivery, by the various involved primary and secondary care providers. Further research should focus on the coordinating role of coaches and find means to overcome current barriers to integrated care delivery. Moreover, we call for more evaluations of similar coaching interventions using real-life data, with a prospective randomized controlled study design to validate and enrich current findings.

## 5. Conclusion

This study shows that implementing out-of-hospital coaching by experienced respiratory nurses is feasible in terms of costs-effectiveness, implementation success, and recipient acceptability. Patients benefited from personal attention, practical advice, exercise training, and motivational meetings, and improvements were seen in various aspects including nutritional intake, dealing with anxiety, and other disease-coping aspects. These improvements are likely to reduce the likelihood of future exacerbation-related hospitalizations and the associated care costs. Furthermore, the qualitative evaluation shows that allocating additional time for disease management education enriches caregivers’ work and enables their professional development. Motivating and educating COPD patients is potentially the most effective approach to slowing disease progression, reducing anxiety, and improving their quality of life, but finding the best approaches requires elaborate scientific attention to different coaching approaches and settings.

## Data availability statement

The datasets presented in this article are not readily available because anonymity and data protection was stated in participant consent form, which was approved by mentioned regional Medical-Ethical Review Committee. Requests to access the datasets should be directed to BN, a.c.noort@rug.nl.

## Ethics statement

The studies involving humans were approved by the Regional Assessment Committee for Patient-related Research, Leeuwarden, Netherlands. The studies were conducted in accordance with the local legislation and institutional requirements. The participants provided their written informed consent to participate in this study.

## Author contributions

BN, TV, JM, and KA contributed to the conception and design of the study. BN and JM organized the data collection. BN performed the statistical analysis and wrote the first draft of the manuscript. JM and EM provided advice on the data analysis. TV and KA wrote sections of the manuscript. All authors contributed to manuscript revision, and read, and approved the submitted version.

## Supplementary material

Supplementary Material File 1 Study setup and measurements. Overview of the patients’ coaching trajectory: meetings and data collection. Description of questionnaires on patient-reported symptoms, wellbeing, and awareness.

Supplementary Material File 2 Statistical analysis. **(A)** Outcomes of panel linear regression model of questionnaire responses (random-effects model). **(B)** Outcomes of panel linear regression model of hospitalization costs (random-effects model).
