# Supplementary material for: Intensive out-of-hospital coaching for frequently hospitalized COPD patients: a before-after feasibility study
Source: Front Med (Lausanne). 2023 Oct 17;10:1195481. doi: 10.3389/fmed.2023.1195481 (PMC10616861; doi:10.3389/fmed.2023.1195481)
Supplement: Supplementary Material File 1 — Study setup and measurements. Overview of the patients’ coaching trajectory: meetings and data collection. Description of questionnaires on patient-reported symptoms, wellbeing, and awareness. [file Table_1.DOCX]

**Supplementary file 1. Study setup and measurements**

***Overview of the patients’ coaching trajectory: meetings and data collection****.*

| Timing (relative to index hospitalization) | | | | | |
| --- | --- | --- | --- | --- | --- |
|  | Day 0 | Day 3-5 | 1-4 days later | 2 weeks to 6 months later | 7 and 12 months later |
| Action | Inclusion and consent | Introduction meeting | Coaching meeting 1 | Coaching meetings 2-7 | Coaching meetings 8, 9 |
| Location of coaching | Hospital | Hospital | Home | Home | By phone |
| Questionnaires |  | MRC  CCQ  SNAQ | MRC  SGRQ  HADS  PAM | MRC  CCQ  SGRQ*  HADS*  PAM*  SNAQ*  PREM^ | MRC  CCQ  SGRQ^#^  HADS^#^  PAM^#^  SNAQ^#^ |

*Only at meeting 6, ^Only at meeting 7, ^#^ Only at meeting 9.

***Description of questionnaires on patient-reported symptoms, well-being, and awareness***

*Dyspnoea*. The Medical Research Council (MRC) dyspnoea scale is based on a questionnaire that consists of ﬁve statements about perceived breathlessness: grade 1, “I only get breathless with strenuous exercise” to grade 5, “I am too breathless to leave the house”. Patients choose the grade that applies to them. The minimum clinically important difference (MCID) is 1 [27].

*COPD-related health status.* The Clinical COPD Questionnaire (CCQ) contains 10 questions answered on a scale of 0–6. The CCQ covers three subdomains: functional status (four questions), symptoms (four questions), and mental status (two questions). The average scores on each subdomain, as well as the average total score are distributed between ‘stable’ (<1), ‘not entirely stable’ (1,2), ‘unstable’ (2,3) and ‘very unstable’ (>3). A change of 0.4 represents the MCID for an individual patient [28, 29].

*Health impairment and well-being*. The St George Respiratory Questionnaire (SGRQ) consists of 50 items with 76 weighted responses and has three component scores: Symptoms, Daily activity, and Impact (i.e., the impact of the illness such as perceiving the illness as being a nuisance to family and friends). A total score is calculated from the three components. The score range for each component and the total score is 0–100 with a score of 100 indicating maximum disability. An improvement of 4 units is considered the MCID [30].

*Anxiety and depression*. The Hospital Anxiety and Depression Scale (HADS) contains 14 questions answered on a scale of 0-3 and produces separate scores for anxiety and depression (7 questions each). The total score ranges from 0 to 21. A score >8 is a predictor of a clinical diagnosis of anxiety and depression. The MCID is 1.5 [31-33].

*Motivation and self-awareness*. The Patient Activation Measure (PAM) consists of 13 items, each answered on a scale ranging from ‘totally disagree’ to ‘totally agree’ or ‘not applicable’ (scale of 1–4). A total raw score is converted into an activation score (ranging from 0–100). Based on this score, four stages of activation are distinguished, where level 1 indicates little awareness of and motivation for the patients’ own role in disease handling, while level 4 indicates high awareness and motivation. The MCID is 5 points [34, 35].

*Nutritional status*. The Short Nutritional Assessment Questionnaire (SNAQ) aims to determine malnutrition during a consultation between a care professional and patient through three questions: Have you unintentionally lost weight? (More than six kilograms in the past six months: 3 points), or more than three kilograms in the past month: 2 points). Have you experienced a reduction in appetite? (yes: 1 point) Have you used probe feeding or nutritional drinks? (yes: 1 point). Adding up all scores provides total maximum of 5 points. A total score of 2 indicates moderate malnutrition, a score of 3 or more indicates severe malnutrition. The MCID is 1, indicating the need for follow-up action by a nurse or dietician [36].

*Patient experience*. The Patient Reported Experience Measure (PREM), chronic version, assesses the patient’s experience in the domains of communication, advice, guidance, treatment, shared decision-making, collaboration, effectiveness and alignment between professionals, organization (5 point Likert scale). A final score was based on a general satisfaction rating (‘Would you recommend this caregiver to others with a chronic disease?’ on a scale of 0-10, with 10 meaning highly likely) [42].
