# Supplementary material for: Intensive out-of-hospital coaching for frequently hospitalized COPD patients: a before-after feasibility study
Source: Front Med (Lausanne). 2023 Oct 17;10:1195481. doi: 10.3389/fmed.2023.1195481 (PMC10616861; doi:10.3389/fmed.2023.1195481)

**Supplementary file 2. Statistical analysis**

**Supplementary file 2A. Outcomes of panel linear regression model of questionnaire responses (random-effects model)**

**
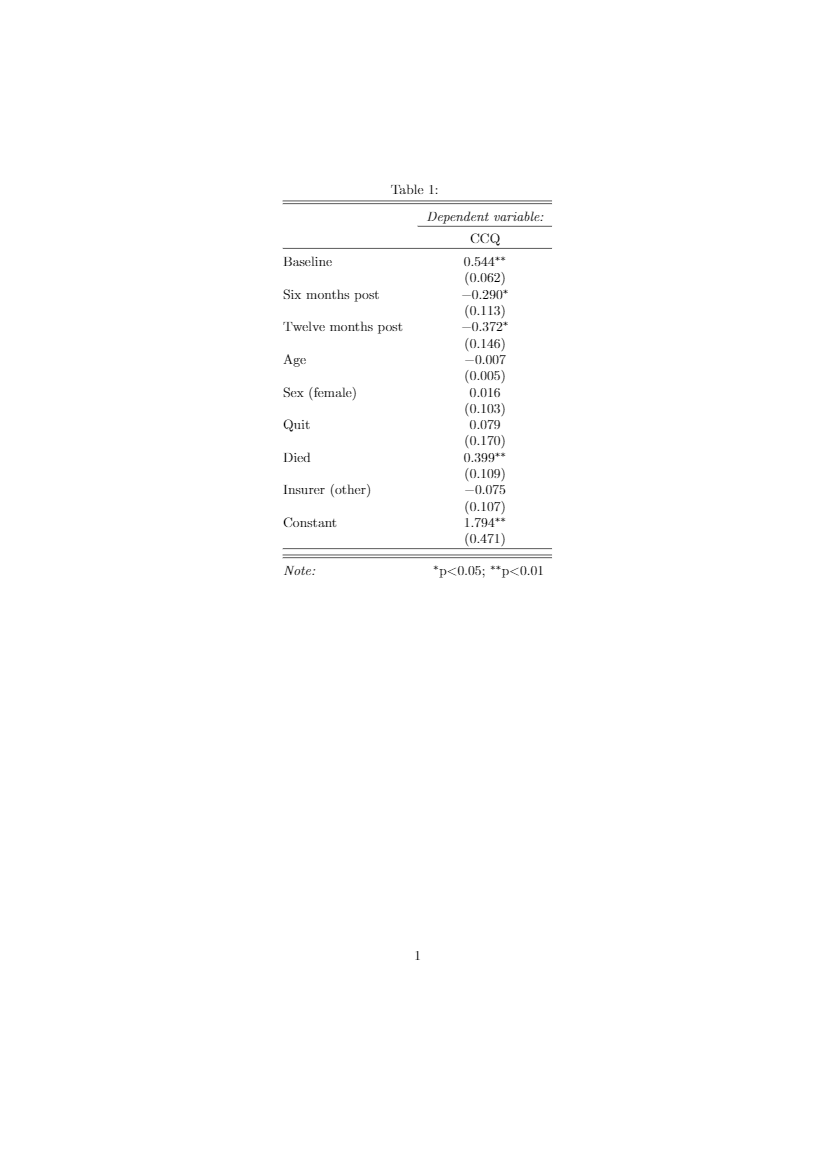
**


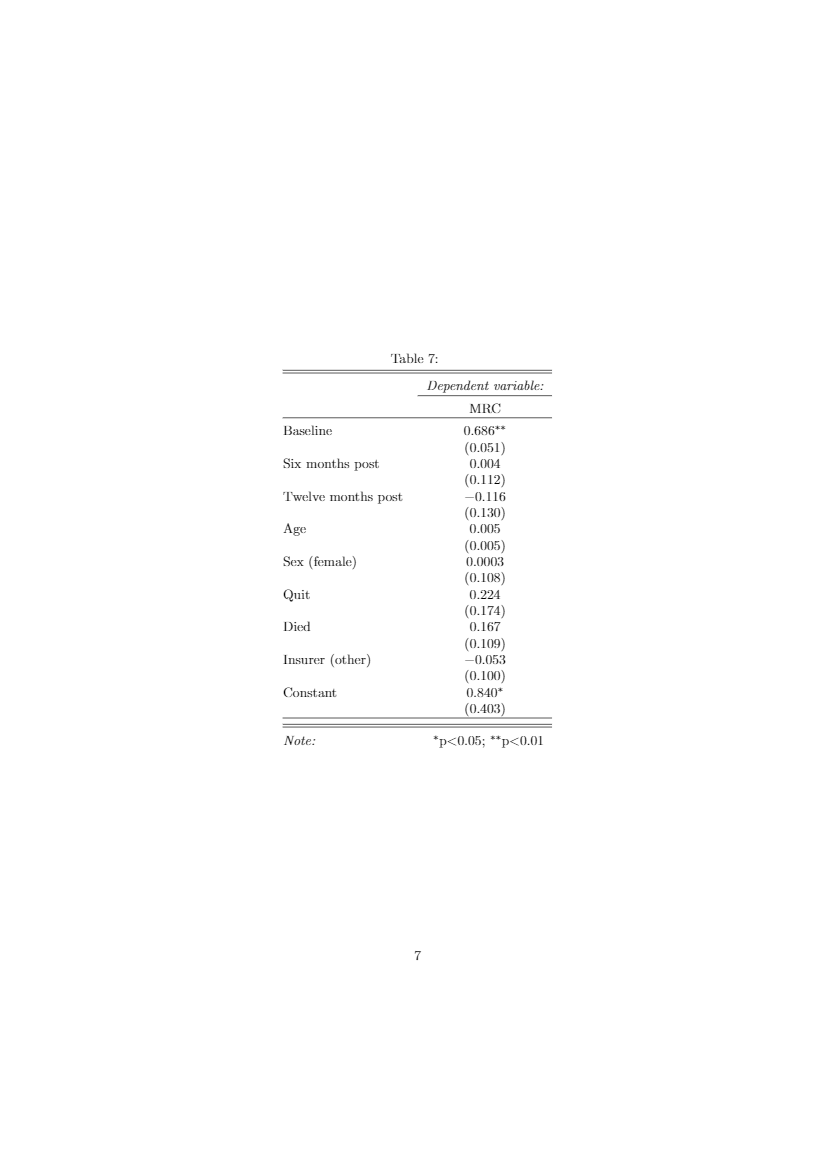

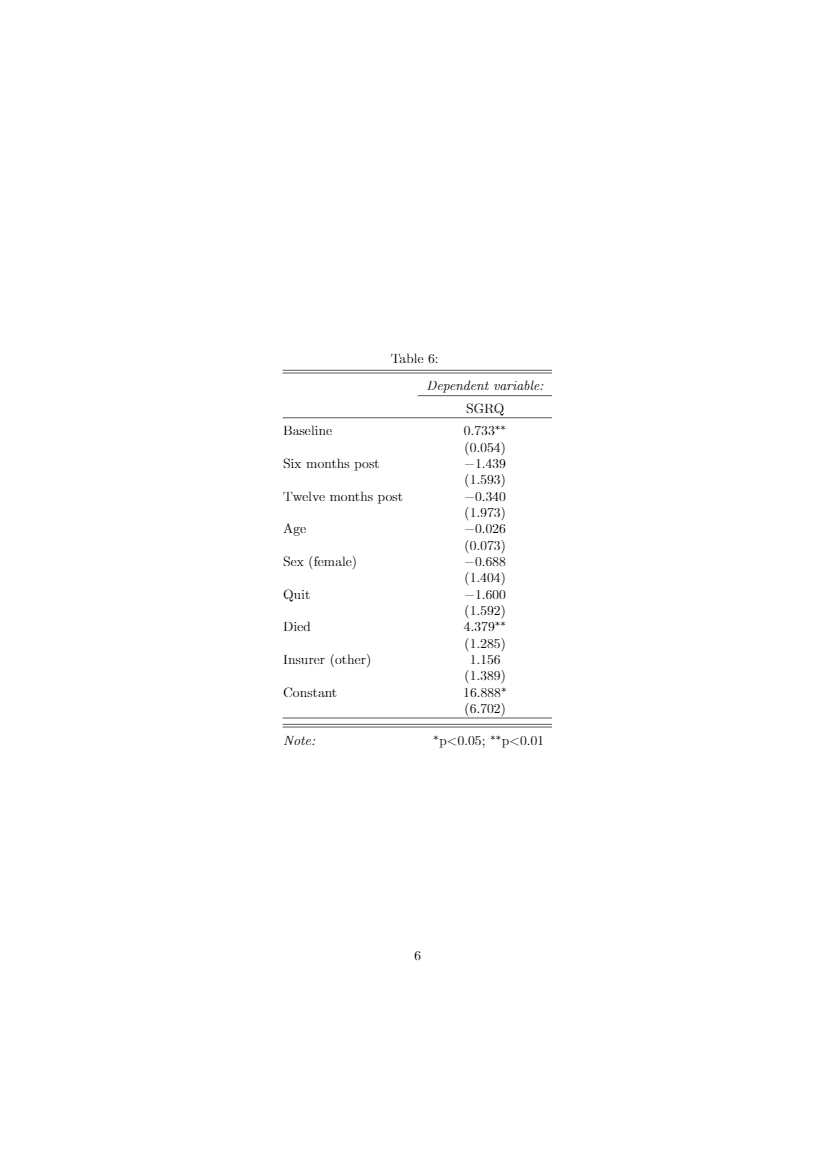

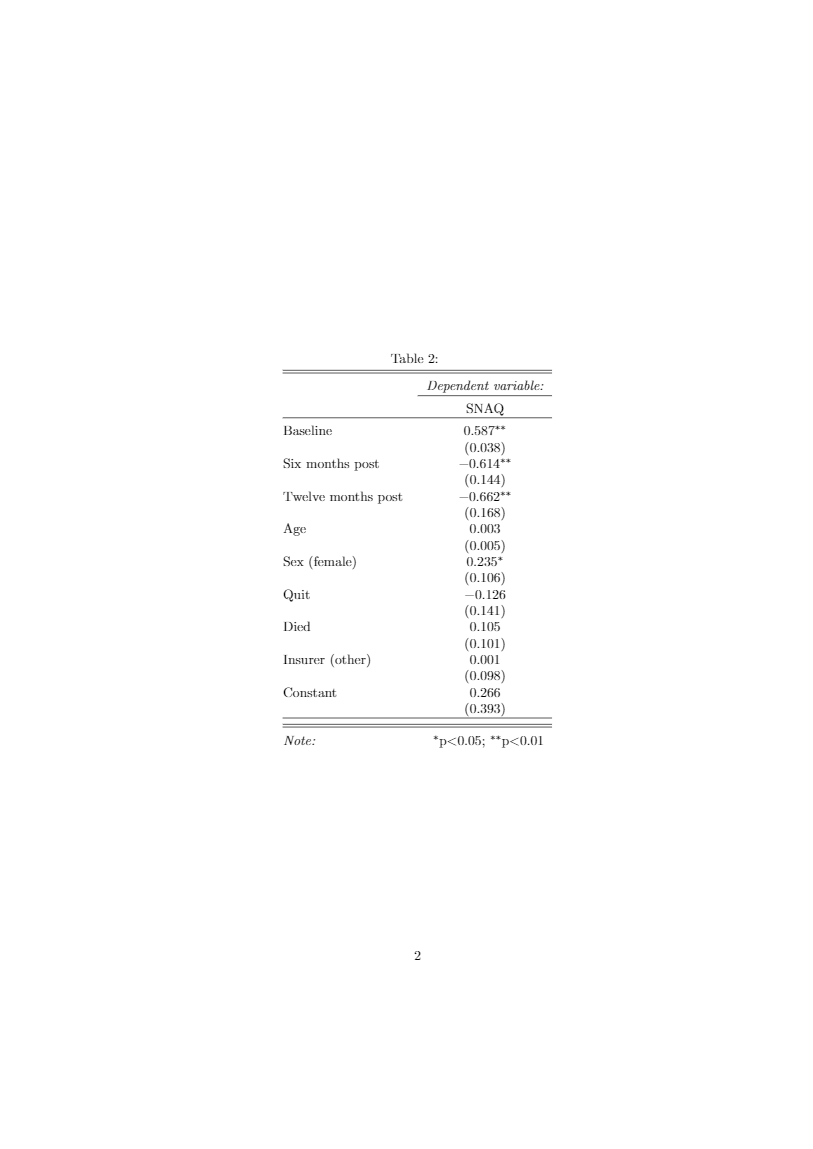


**
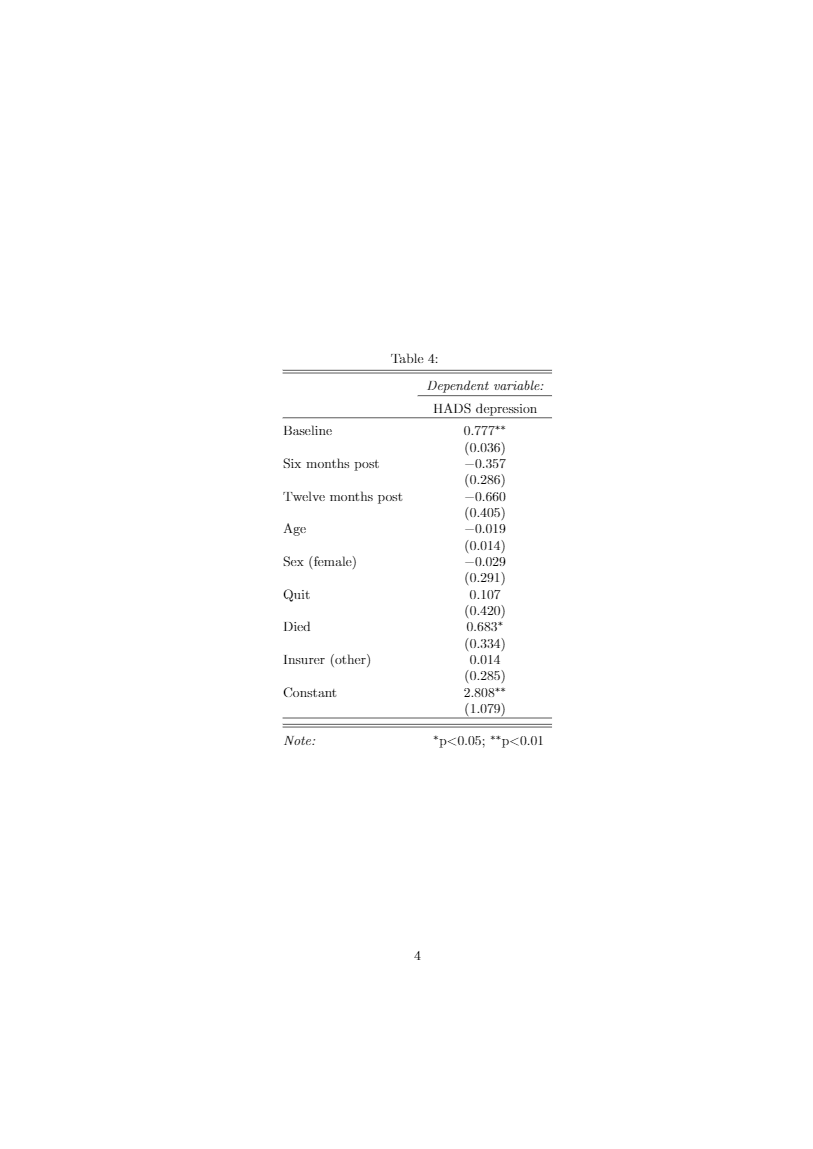

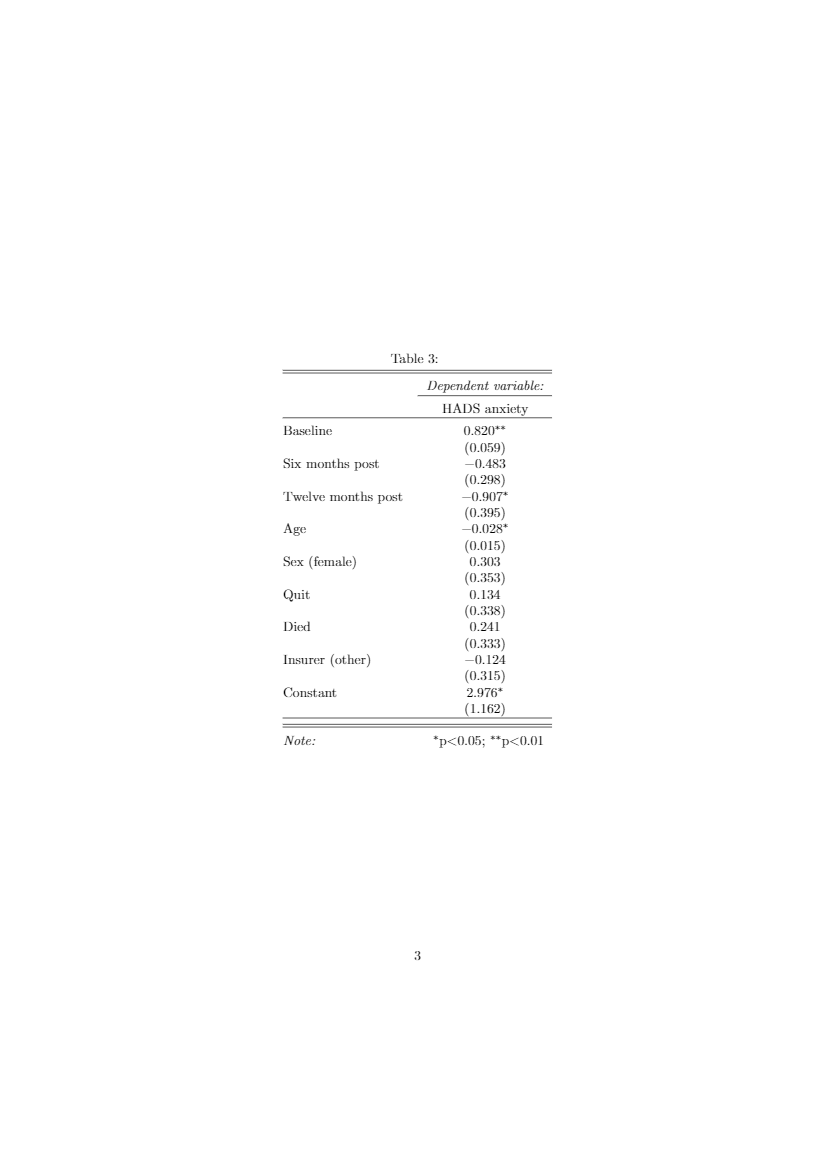
**


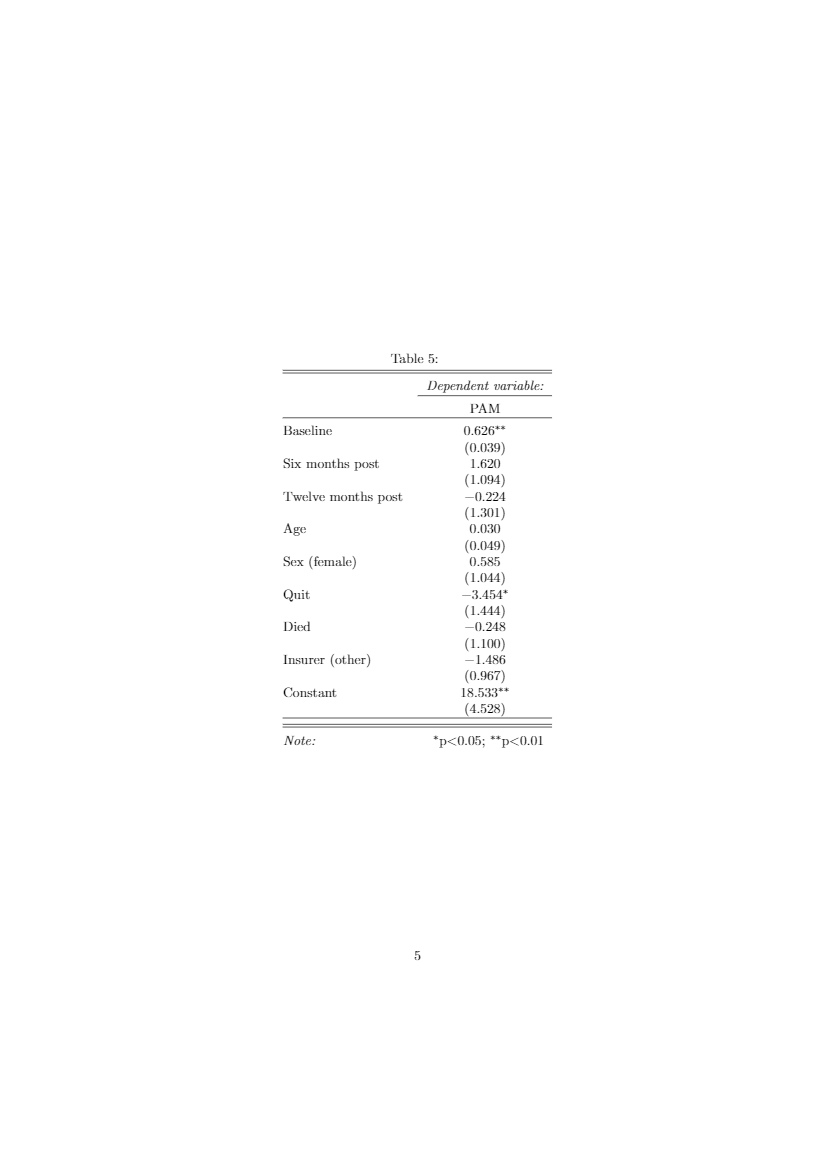


**Supplementary file 2B. Outcomes of panel linear regression model of hospitalization costs and hospitalization rate (random-effects model)**

*Hospitalization costs*





*Hospitalization rate*


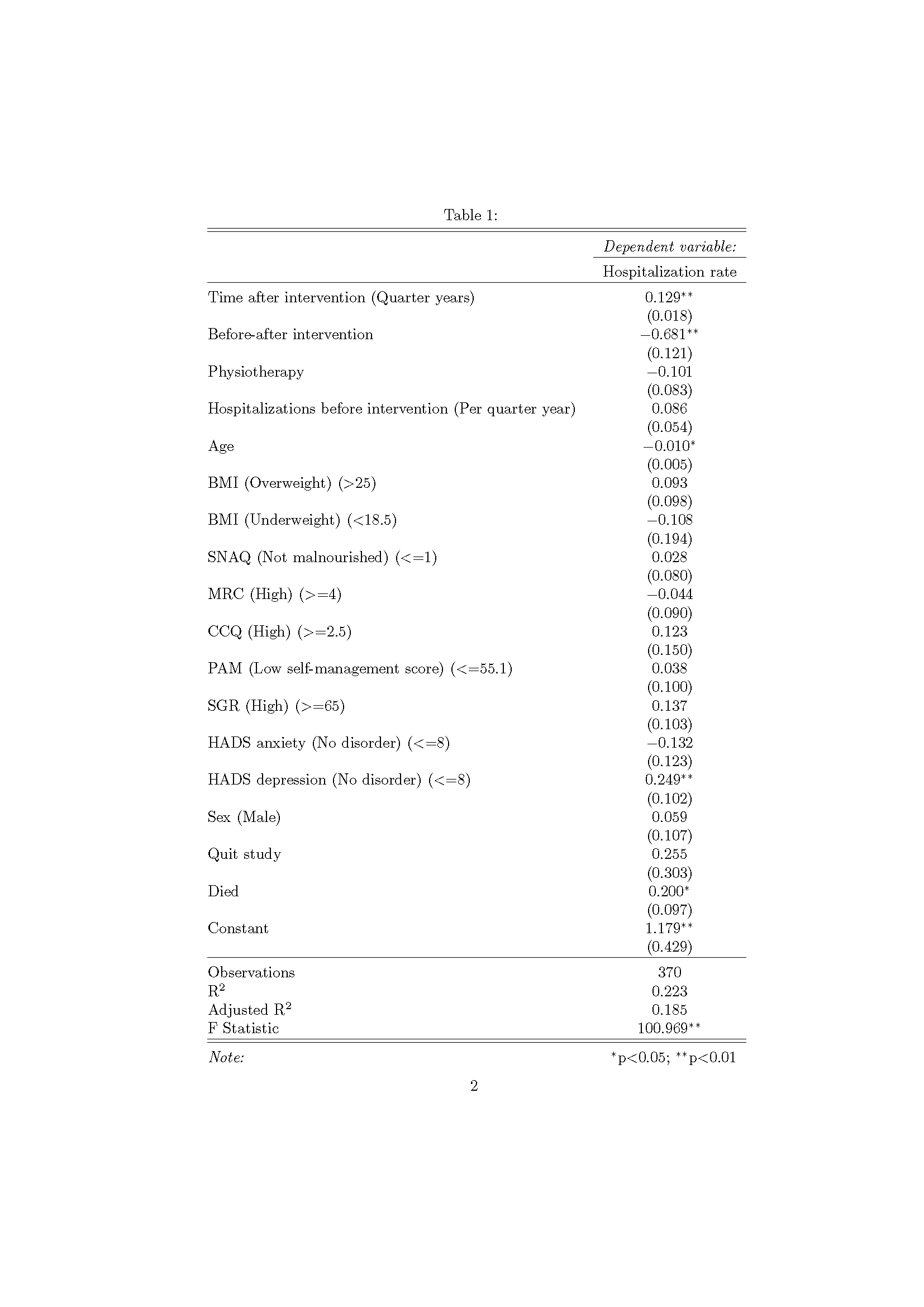

Supplement: Supplementary Material File 2 — Statistical analysis. (A) Outcomes of panel linear regression model of questionnaire responses (random-effects model). (B) Outcomes of panel linear regression model of hospitalization costs (random-effects model). [file Table_2.DOCX]
